# The micro‐shear bond strength of resin cements to aged laser bleached enamel after using different desensitizing agents

**DOI:** 10.1002/cre2.496

**Published:** 2021-09-25

**Authors:** Aya E. Samaha, Ahmad K. ElFadl, Mohammed N. Anwar

**Affiliations:** ^1^ Faculty of Dentistry Ain Shams university Cairo Egypt

**Keywords:** bleaching, desensitizing agent, fluoride, laser, resin cement

## Abstract

**Objectives:**

To evaluate the micro‐shear bond strength of two resin cements to aged laser bleached enamel after the application of three different desensitizing agents.

**Materials and methods:**

Forty extracted human central and lateral incisors were prepared and bleached using laser activation bleaching protocol. The teeth were assigned randomly into four groups for desensitization; G1: No post‐bleaching treatment, G2: GC MI Paste Plus, G3: Hydroxyapatite nanoparticles (n‐HAP) and G4: Flor‐Opal. Specimens were subjected to aging for 6 months. All groups were subdivided into two subgroups according to the resin cements used (dual‐curing resin cement and light‐curing resin cement).

**Results:**

Flor‐Opal groups showed the highest statistically significant micro‐shear bond strength (MSBS), followed by GC MI Paste Plus and n‐HAP groups with no statistically significant difference between them. The light‐curing resin cement had statistically higher MSBS than dual‐curing resin cement in case of no‐post bleaching treatment and n‐HAP groups, and no statistical difference in case of GC MI Paste Plus and Flor‐Opal groups.

**Conclusion:**

Usage of desensitizing agents containing, CPP‐ACP, n‐HAP or fluoride after laser bleaching can enhance the bond strength of the resin cements to enamel.

**Clinical significance:**

The composition of the desensitizing agents applied after laser bleaching could interfere in bond strength values.

## 1 INTRODUCTION

Laser bleaching is becoming an increasingly common conservative and noninvasive procedure in dental clinic for patients seeking a more attractive smile. The application of diode laser at both wavelengths (810 and 980) had proven to reduce the enamel surface alternation (Azarbayjani et al., 2018) and surface roughness (Anaraki et al., 2015) that happen during bleaching. The process of bleaching consists of a multipart oxidation course initiated by the bleaching gel, releasing reactive oxygen species, breaking down organic pigment molecules double bonds and producing smaller, and clearer compounds (Kihn, 2007). These molecules are small enough to diffuse out of the tooth or absorb less light and hence leading to a lighter tooth appearance (Sulieman, 2008). Despite the quick and immediate results of the bleaching treatment, nearly 70% of the patients would suffer from tooth sensitivity (Cartagena et al., 2015). Multiple morphological changes of enamel and considerable mineral loss (Al‐Salehi et al., 2007) were observed, in addition to the reduction of the bond strength (Dishman et al., 1994; Garcia‐Godoy et al., 1993; Titley et al., 1993) up to 3 weeks after bleaching (Miyazaki et al., 2004).

Trying to overcome the sensitivity post operatively, several desensitizing agents have been introduced for use before or after bleaching or in association with bleaching gels (Nanjundasetty & Ashrafulla, 2016). Moreover, such desensitizing agents can reduce the morphological changes (Coceska et al., 2016) and the loss of mineral content (Sasaki, 2015) in the enamel and regain its dropped microhardness (Samaha & Gomaa, 2020) after bleaching. New technologies are being introduced in this field such as the casein phosphopeptide‐amorphous calcium phosphate (CPP‐ACP) which provides a calcium and phosphate reservoir that can bind to enamel surface (Lata et al., 2010) to avoid the negative changes of bleaching to enamel. Recently, for improving enamel remineralization, synthetic nano‐hydroxyapatite has been well thought out for enamel repair (de Carvalho et al., 2014). In addition, the topical fluoride application; that is being used alone or in combination with other elements, had proven its effectiveness to remineralize bleached enamel, increase its microhardness and decrease its mineral loss (Bizhang et al., 2006; Borges et al., 2010; Chen et al., 2008).

Combined esthetic interventions sometimes are required for patients who are not satisfied with the bleaching results or whose cases require change of form and shape. Veneering has become a reliable technique used by dentists to meet the patients' esthetic needs while conserving the remaining tooth structure. However, 12.5% of veneers' failure (Davidowitz & Kotick, 2011) is due to the improper selection or application of the adhesive or the resin cement (Bona & Kelly, 2008). Resin cements may be classified according to their polymerization mechanisms into light‐curing, chemical‐curing, and dual‐curing. In general, light curing resin cements are favored by dentists for cementation of laminate veneers due to their color stability and longer working time in comparison to both dual and chemical‐curing resin cements (Oztürk et al., 2012). Changes in the surface properties of enamel due to bleaching are likely to have an impact on the long‐term success of the restoration, therefore, the interaction of bleaching followed by desensitization with any subsequent dental procedure should be considered.

Although there are many studies on bond strength of composite resin to bleached enamel, there is not adequate information about the bond strength between resin cements used for luting ceramic laminate veneers and bleached desensitized enamel after aging. Very few studies have examined whether the composition of the desensitizing agents applied after laser bleaching could interfere in bond strength values. The null hypotheses for this study were as follows: (i) There is no difference in the micro‐shear bond strength (MSBS) of two different resin cements to aged laser bleached enamel; (ii) The type of the desensitizing agents used after bleaching do not affect MSBS values.

## 2 MATERIALS AND METHODS

### 2.1 Enamel specimens' preparation:

Forty freshly extracted sound human upper anterior incisors were extracted due to therapeutic reasons and were collected from the Oral and Maxillofacial Department, Faculty of Dentistry, Ain‐Shams University to be used in this study. Teeth were examined under light stereomicroscope (Steeozoom 5, Bausch & Lomp) to exclude teeth with cracks, abrasions, or decay. Teeth were cleaned, disinfected and stored in fresh, renewed deionized water at 4°C.

Teeth were sectioned 2 mm below the cemento‐enamel junction using diamond disks (MANI, Inc., Japan) under copious water spray coolant in the Laboratory of Department of Oral Biology, Faculty of Dentistry, Ain‐Shams University. Each specimen, with the labial surface facing downward, was positioned in fast set chemical curing acrylic resin (Acrostone, Egypt) in polyvinyl chloride rings (PVC) of 19 mm diameter. After hardening, the enamel surfaces were flattened using silicon carbide paper grits #320 and #400 and polished with #600, #1200, and #2400 grit using wet aluminum oxide abrasive papers for 30 s for each in a circular motion. Any specimen that showed dentin under magnification was discarded. The specimens were ultrasonically cleaned for 5 min.

### 2.2 Bleaching procedure

Enamel surfaces were bleached with 35% hydrogen peroxide bleaching gel (Heydent, GmbH, Kaufering, Germany) which was activated using Photon Plus 980 nm Zolar diode laser (Zolar Technology & Manufacturing Co. Inc., Mississauga, ON, Canada). The bleaching gel was squeezed until a homogenous colored mass of the gel was obtained. A uniform layer of the bleaching gel was placed on the enamel surface of each specimen, followed by laser irradiation, in a continuous mode at 7 W power for 30 s according to the manufacturer's recommendation. Before laser irradiation, the output power was measured by Gentec powermeter (Gentec electrooptique, Inc., QC, Canada). The gel was rinsed off the enamel surfaces using running water for 30 s.

### 2.3 Specimens' grouping and desensitization protocol

The specimens were divided randomly into four groups (*n* = 28) according to the post‐bleaching treatment: G1: No post‐bleaching treatment, G2: GC MI Paste Plus, G3: Hydroxyapatite nanoparticles and G4: Flor‐Opal. The materials used in the study are listed in Table 1.

**Table 1:** Materials used in the study

Material	Code	Composition	Manufacturer	Batch no.
GC MI Paste Plus	G2	Glycerol, 5–10% CPP–ACP, pure water, zinc oxide, CMC–Na, xylitol, d‐sorbitol, silicon dioxide, phosphoric acid, titanium dioxide, guar gum, sodium saccharin, ethyl‐p‐hydroxybenzoate, magnesium oxide, propylene glycol, butyl‐p‐hydroxybenzoate, propyl‐p‐hydroxybenzoat. Fluoride level is 0.2% wt/wt (900 ppm).	GC Corp., Tokyo, Japan.	090813M
Hydroxyapatite nanoparticles	G3			
Flor‐Opal	G4	0.5% fluoride ion pH 6.5	Ultradent, Inc., South Jordan, USA.	F115
DUO‐LINK universal resin luting cement	DC	Base: UDMA, Bis‐GMA, TEGDMA, fiberglass Catalyst:10–30% Bis‐GMA, 1% dibenzoyl peroxide	BISCO, Schaumburg, USA.	1900003959
Choice 2 light cured resin cement	LC	UDMA, BisGMA, tetrahydrofurfuryl methacrylate, glass strontium, amorphous silica	BISCO, Schaumburg, USA.	1900004426

Abbreviations: Bis‐GMA, Bisphenol A‐glycidylmethacrylate; CMC‐Na, sodium carboxymethyl cellulose; TEGDMA, tetraethyleneglycol dimethacrylate; UDMA, urethane dimethacrylate.

G1 had no post‐bleaching treatment while, the desensitizing agents in G2 and G4 were applied in a uniform layer on the enamel surface of the specimens for 5 and 30 min, respectively, according to the manufacturer's instructions. As for G3, n‐HAP was prepared by Nanotech Company for photo‐electronics (dreamland, 6th October, Egypt). It was set using wet chemical method as stated by (Jarcho et al., 1977; Yubao et al., 1994) of calcium nitrate with ammonium hydroxide ([NH4]₂ HPO4). The final product was a white powder with rod shape particles (as seen under TEM) and average size of L 90 ± 10 nm, W 20 ± 5 nm where the grain size was controlled by changing the time and the temperature of HA precipitation, with pH values between 10 and 12 and the reaction was performed at room temperature. The prepared paste was applied on the specimen followed by scrubbing using a microbrush for 10s and then it was left undisturbed for 4 min.

All specimens, including G1, were stored for 6 months in artificial saliva at 37°C, which was replenished every day. The artificial saliva composed of 1.5 mmol/L calcium chloride, 8.2 mmol/L sodium bicarbonate, 4.8 mmol/L sodium chloride, 137 mmol/L potassium chloride, 4 mmol/L potassium dehydrogen phosphate, and 100 ml deionized water.

### 2.4 Bonding

Prophylaxis of the bonding area was performed with pumice stone and a brush. The specimens were etched with 37% phosphoric acid etching gel for 15 s (Pentron Clinical, Orange, CA, batch no. 671065) then were rinsed and dried for the same length of time, leaving the enamel visibly moist. The adhesive system recommended by the same manufacturer was used in this study (All Bond Universal, BISCO, Schaumburg, batch no. 1900004262). The adhesive was applied and scrubbed by a micro‐brush in two coats for 10–15 s per coat according to the manufacturer's instructions. It was then gently air‐dried, and light activated for 10 s (Optilux, Demetron; Orange, CA) with a power density of 600 mW/cm^2^.

Each group was then randomly assigned to two subgroups (*n* = 14) according to the two resin cements used. The resin cements were filled on all the treated enamel surfaces using Teflon tubes of approximately 0.75 mm internal diameter and 1 mm height. The tubes were photo activated one at a time for 40 s each to achieve final set. In order to apply a pressure on the cement, simulating finger pressure during cementation of a crown, a glass slab was applied on the tubes and loaded with 4N. The Teflon tubes were then dismantled to yield the samples and any excess adhesive was scratched using a lancet.

### 2.5 Bond strength testing and failure analysis

The MSBS test was carried out after 24 h, using a universal testing machine (Instron 8500, England) with a load cell of 10 KN. A knife‐edge rod with a width of 0.5 mm was applied at the interface of the resin cement with the enamel at cross‐head speed of 0.5 mm/min. The debonded enamel sites were photographed using a camera (Nikon WAT 221S, Japan) and a stereomicroscope (Nikon SMZ 745T, Japan) at ×40 magnification to determine the modes of bond failure. Failure modes were classified as adhesive, cohesive, or mixed and were defined as follows: Adhesive failure showed more than 70% of enamel surface or resin cement exposure, cohesive failure showed more than 70% failure in enamel or resin cement and mixed failure showed both failures.

### 2.6 Statistical analysis

Collected data was subjected to Shapiro–Wilk test which indicated normally distributed MSBS results (*p* > 0.05). Two‐way analysis of variance (ANOVA) followed by Tukey HSD post hoc test were performed to evaluate the effect of the desensitizing protocol, the type of resin cement and the interaction between them on MSBS. One‐way ANOVA followed by Tukey HSD post hoc test were performed to evaluate the effect of the desensitizing protocol within the same type of resin cement on MSBS. Independent *t* test was carried out to evaluate the effect of the resin cement type within the same desensitization protocol on MSBS. A *p*‐value of <0.05 was considered as statistically significant.

## 3 RESULTS

### 3.1 Micro‐shear bond strength test and failure analysis

**Figure 1:**
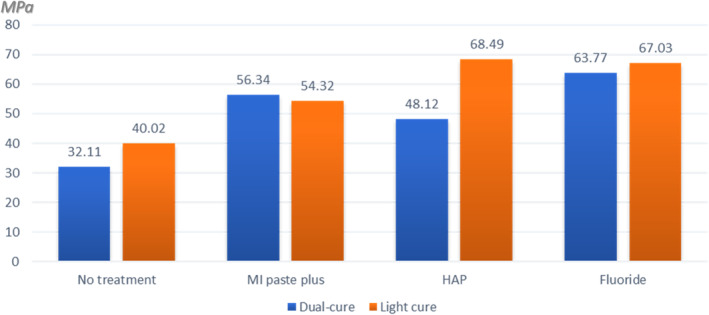
Bar graph showing the mean micro‐shear bond strength of the resin cements with different desensitizing protocols

Two‐way ANOVA test for MSBS revealed that the type of desensitization, the type of resin cement and the interaction between them have statistically significant effect (*p* < 0.0001, *p* < 0.01, and *p* < 0.001, respectively). Tukey HSD post hoc test showed that for desensitization protocol regardless to the type of the resin cement; Flor‐Opal groups had the highest statistically significant MSBS, followed by GC MI Paste Plus and n‐HAP groups with no statistically significant difference between them. The no‐post bleaching treatment groups showed the least MSBS (Table 2). One‐way ANOVA followed by Tukey HSD post hoc test for the desensitization protocol for the same resin cement revealed that; first, regarding dual‐curing resin cement, Flor‐Opal group showed the highest statistically significant MSBS with no statistical difference to GC MI Paste Plus group. On the other hand, n‐HAP group showed statistically significant lower MSBS than Flor‐Opal group with no difference with GC MI Paste Plus group. Second, regarding light‐curing resin cement, Flor‐Opal and n‐HAP groups showed the highest statistically significant MSBS with no difference between them, followed by GC MI Paste Plus group, while the no‐post bleaching treatment groups showed the least MSBS. Independent *t* test for the mean difference between dual‐curing and light‐curing resin cement MSBS for the same desensitization protocol showed that light‐curing resin cement had statistically higher MSBS than dual‐curing resin cement in case of no‐post bleaching treatment and n‐HAP groups (*p* = 0.002 and *p* = 0.004, respectively). There was no statistical difference between light and dual‐curing resin cement in case of GC MI Paste Plus and Flor‐Opal groups (Table 3, Figure 1).

**Table 2:** The mean MSBS of the 2 resin cements for the same desensitization protocol

Desensitization	No‐post bleaching treatment (G1)	GC MI Paste Plus (G2)	n‐HAP (G3)	Flor‐Opal (G4)	*p* Value
	36.06 ± 5.7 ^c^	55.29 ± 6.3 ^b^	58.71 ± 7.1 ^b^	65.42 ± 6.5^a^	0.0001
Resin cement	Dual‐curing resin cement		Light‐curing resin cement		*p* Value
	49.78 ± 8.3 ^b^		58.68 ± 7.9 ^a^		0.01

*Note*: Different lower‐case superscript letters within the same row indicate statistically significant difference *p* < 0.05.

**Table 3:** The mean MSBS of the resin cements with different desensitizing protocols

Desensitization	No‐post bleaching treatment (G1)	GC MI Paste Plus (G2)	n‐HAP (G3)	Flor‐Opal (G4)	*p* Value
Dual‐curing resin cement	32.11 ± 5.8 ^c^	56.34 ± 11.2 ^ab^	48.12 ± 16.9 ^b^	63.77 ± 11.9 ^a^	0.0001
Light‐curing resin cement	40.02 ± 6.4 ^c^	54.32 ± 9.9 ^b^	68.49 ± 12.6 ^a^	67.03 ± 14.5 ^a^	0.0001
*p* Value	<0.002*	<0.625 ns	<0.004*	<0.554 ns	

*Note*: Different lower‐case superscript letters within the same row indicate statistically significant difference *p* < 0.05.

^*^ Within the same column indicates statistically significant difference *p* < 0.05.

### 3.2 Failure analysis

**Figure 2:**
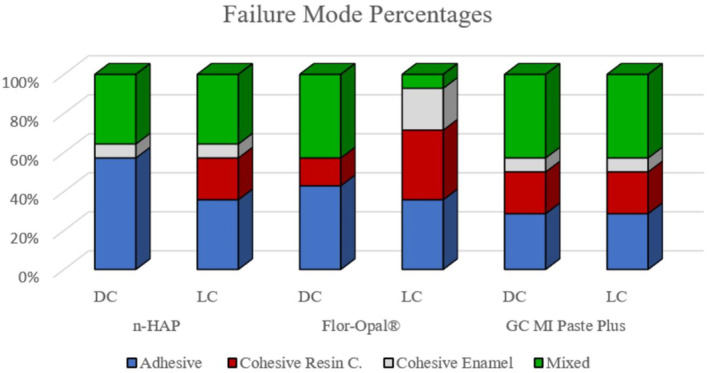
Bar chart showing the failure mode percentages in each group

**Figure 3:**
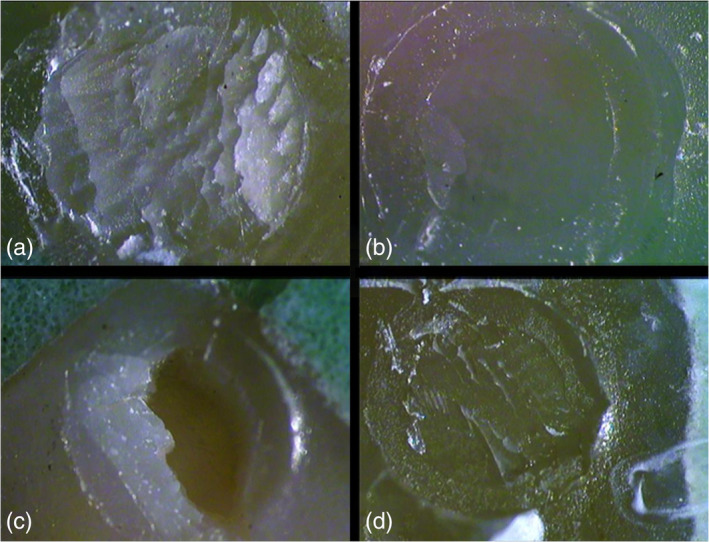
Stereo‐microscope photograph of the representative specimens for different modes of failure; (a) cohesive failure in the resin cement, (b) adhesive failure, (c) cohesive failure in the enamel, (d) mixed failure

The distribution of failures after the MSBS test within the different tested groups are presented in Table 4. It was noticed that most of the adhesive failures in this study were in the n‐HAP group with the dual‐curing resin cement. The modes of failure of the different tested groups in percentage are represented in Figure 2. Stereo‐microscope photograph of the representative specimens for different modes of failure are represented in Figure 3.

**Table 4:** The modes of failure of the different tested groups

		Adhesive failure	Cohesive failure (resin cement)	Cohesive failure (enamel)	Mixed failure
GC MI Paste Plus	DC	4	3	1	6
	LC	4	3	1	6
n‐HAP	DC	8	0	1	5
	LC	5	3	1	5
Flor‐Opal	DC	6	2	0	6
	LC	5	5	3	1

## 4 DISCUSSION

During our clinical practice, we can face the problem of non‐lasting bleaching results where the patient refuses to go through bleaching again and is seeking a more permanent outcome or where bonded restorations are required to overcome some esthetic deficiencies. Dealing with such problems might require the change of the treatment plan and combining a totally conservative approach to a restorative approach. Laminate veneers can become the dentist's second choice when it comes to anterior discoloration, which brings us to the necessity to assess the bond strength of resin cements to enamel after being desensitized and aged. The bonding of resin cements to desensitized aged laser bleached enamel in the present study showed increased bond strength than when the cements were bonded to undesensitized surfaces. The enamel specimens in this study were flattened in order to obtain a suitable surface for bonding and bond testing. In addition, the removal of the highly mineralized fluorapatite superficial layer was mandatory as it can vary from one patient to another. Intraorally, the laminate veneers are subjected to complex displacing stresses that can be measured as tensile or shear forces (Shimada et al., 2002). Therefore, the MSBS testing was performed in this study to measure the bond strength of the resin cements to the enamel after different desensitizing protocols as it allows a more uniform distribution of stresses due to its small cross‐section and the usage of less number of natural teeth (Armstrong et al., 2010).

The power bleaching method used in this study was the diode laser irradiation at 980 nm wavelength as it showed abilities to prevent the adverse effects of bleaching on the enamel surface (Anaraki et al., 2015; Azarbayjani et al., 2018). It was suggested that the laser‐bleached enamel demonstrates an increase in the size of apatite crystals or in their crystallinity due to the removal of proteins attached to apatite plates (Azarbayjani et al., 2018). In addition, the bleaching efficiency of the diode lasers is high due to the ability of chromophores of the laser‐activated gels to absorb the laser narrow wavelength, which is translated into less heat production (Anaraki et al., 2015).

A 35% hydrogen peroxide bleaching gel was used, as it can undergo several chemical breakdowns, releasing free radicals, reactive oxygen ions, and peroxide anions (Dahl & Pallesen, 2003) which itself can oxidize the organic and inorganic materials, including chromophores (Joiner, 2006). Several studies have reported the adverse effect of the remaining oxygen in the enamel prisms leading to a significant reduction of the bond strength of resin‐based composite immediately after bleaching (Bittencourt et al., 2010; Dishman et al., 1994; Kwon, 2011) and showed that the formed resin tags are shorter, less defined and fewer in number (Bittencourt et al., 2013). Others recommended duration up to 7 days before bonding to bleached enamel (Bittencourt et al., 2010; Britto et al., 2015; Dishman et al., 1994; Unlu et al., 2008), on the other hand, higher bond strength values where observed after 21 days (Bittencourt et al., 2010; Kwon, 2011). This was attributed to the fact that the immersion in water or saliva can cause the residual oxygen to lose its activity and dilute its effect on the enamel (Titley et al., 1993). However, Perdigao et al. (1998) concluded that there was no oxygen difference between the bleached and non‐bleached enamel, suggesting that the reduction of the bond strength may be due to the structural micromorphological alterations to the tissues, where bleaching can cause alteration of the calcium/phosphate ratio (Al‐Salehi et al., 2007; Coceska et al., 2016; Llena et al., 2018) associated with massive reduction in the surface microhardness (Attin et al., 2007; Borges et al., 2010). In our study, this explains G1 results that showed the lowest MSBS in comparison to all other groups.

Regarding our study, an aging period of 6 months was applied on all specimens with complete insurance that the difference in bond strength will be attributed to the chemical composition of the resin cement and its interaction with the altered enamel surface.

Considering the earlier mentioned facts, in addition to the postoperative hypersensitivity, the application of a desensitizing agent with remineralizing potentials after bleaching has become a known protocol that is recommended by manufacturers, though, this process would leave us with an altered enamel surface. It has been reported that CPP‐ACP application, after in‐office bleaching, is capable to avert negative changes of roughness and hardness to enamel (Ata, 2019; Gama Cunha et al., 2012; Shadman et al., 2015) and increase the calcium levels of enamel (Llena et al., 2019; Samaha & Gomaa, 2020). Its action depends on maintaining high concentration of calcium and phosphate ions and localizing ACP at the tooth surface (Coceska et al., 2016; de Carvalho et al., 2014) which interact with carbonates to block surface defects with hydroxyapatite crystals (HA; Moule et al., 2007), increasing the density of HA crystals on the surface (Khoroushi et al., 2011). The addition of fluoride to the gel has shown to enhance its remineralizing effect (Borges et al., 2010). According to our results, G2 showed a statistical significance in relation to the control group, which coincides with several studies even when the fluoride was not incorporated (Elzuhery et al., 2013) and it showed lower results in comparison to G4, this might be due to the protective effect which is efficiently provided by the CPP‐ACP products compared to fluoride‐ only containing products (Poggio et al., 2016), leaving an acid‐resistant layer on the enamel surface, making it more resistant to any acidic challenge (Moule et al., 2007).

Showing beneficial effect on the remineralization of bleached enamel (Ata, 2019; Coceska et al., 2016), n‐HAP was used in this study. GC MI Paste Plus and n‐HAP showed similar repair results (Rahiotis & Vougiouklakis, 2007; Shadman et al., 2015) which coincide with our results with the dual‐curing resin cement. The remineralization of enamel by n‐HAP might be due to the rod‐like crystal structure of the n‐HAP (Wu et al., 2013) and its ability to strongly adsorb to the bleached enamel surface under in vitro conditions with a size of 20 nm which can fit well with the nano‐defects caused by enamel surface erosion (Li et al., 2008), increasing its microhardness (Heshmat et al., 2016). However, the n‐HAP prepared in this study varied from L 90 ± 10 nm and W 20 ± 5 nm, which might have led to the decrease of its precipitation in comparison to the Flor‐Opal.

This study demonstrated that the specimens submitted to bleaching followed by the application of Flor‐Opal presented a significantly higher bond strength compared to nondesensitized teeth and all the other groups. Assessment of failure mode showed predominance of the cohesive and mixed failure modes; thereby confirming that fluoride positively influenced the bond strength of resin cements to enamel and showed the highest MSBS among all groups. Topical application of fluoride has showed to be effective in regaining the bleached enamel microhardness after 28 days (Borges et al., 2010; Chen et al., 2008) and attain a high bond strength values after 14, hydrogen peroxide was able to open diffusion channels in the enamel which facilitated the transmission of fluoride into deeper enamel layers, and hence increasing remineralization (Klimek et al., 1982). It is also suggested that the fluoride ions released from desensitizing agent could promote formation of fluoridated apatite on tooth surface (Britto et al., 2015). However, regarding light‐curing resin cement, Flor‐Opal and n‐HAP groups showed the highest statistically significant MSBS in comparison to the rest of the groups. A study conducted on toothpastes remineralization capability, toothpastes containing n‐HAP revealed higher remineralizing effects compared to amine fluoride toothpastes with bovine dentin and enamel, which was correlated to the higher pH values of the n‐HAp slurries in comparison to the fluoride slurries (Tschoppe et al., 2011).

Controversial to our study results, several studies had reported reduction in the bond strength to enamel after bleaching/desensitizer application. Metz et al. (2007) performed an in vivo study to find out that bleaching/desensitizer (2.26% fluoride+ potassium nitrate) regimen led to decrease in the bond strength up to 14 days. Another study reported that use of carbamide peroxide and CPP‐ACP significantly reduced bond strength up to 3 days (Moule et al., 2007). Khoroushi et al (Khoroushi & Ghazalgoo, 2013) reported that bleaching/desensitizer treatment (Relief ACP) significantly decreased bond strength of composite resin to enamel up to 2 weeks. The short post bleaching time before bonding; which was avoided in our study, can explain these results according to Bittencourt et al. (2013)) who speculated that the restoration time factor and not the topical desensitizer application was actually responsible for the increase in the bond strength values. In addition, our specimens were stored for 6 months in artificial saliva which is responsible for the remineralization of bleached enamel (Oltu & Gurgan, 2000; Unlu et al., 2008; Uysal et al., 2003), where the micro‐surface defects created by bleaching provided suitable sites for high mineral content ion deposition, similar to that of the arrested caries (Heshmat et al., 2016).

Among the studies which are in agreement with our present study, Keçik et al. (2008) with acidulated phosphate fluoride and CPP‐ACP and Xiaojun et al. (2009) with CPP‐ACP Paste which both did not show a decrease of bond strength to enamel. Shadman et al. also applied CPP‐ACP to enamel and observed no reduction in the shear bond strength with an etch and rinse adhesive (Shadman et al., 2015).

Since the etch and rinse adhesives provide higher bond strengths than the self‐etching adhesives with bleached enamel (Gurgan et al., 2009), therefore, the same compatible two‐step etch and rinse adhesive was used for both resin cements to ensure that the difference in the results are related to different chemical composition of the two resin cements. According to our results, there was no statistical difference between light and dual‐curing resin cement in Flor‐Opal and MI Paste Plus groups. Concluded by several studies, the effect of the resin cement type on shear bond strength is much lower as long as the type of preparation surface is constant (Hikita et al., 2007; Nada et al., 2016) especially that regardless to the type of the resin cement; Flor‐Opal groups had the highest statistically significant MSBS, followed by MI Paste Plus. Yet, regarding the n‐HAP and no treatment groups, the light‐curing resin cement had statistically higher MSBS than dual‐curing resin cement. The cements composition used varied in the polymerization, volumetric shrinkage, film thickness, and size of filler, such difference can affect the shear bond strength of the materials.

The relation between shear bond strength and failure mode was explained in literature (Peumans et al., 2016) where the cohesive failure of cements is always correlated with high shear bond strength values and that the mixed failure is more favorable than the adhesive failure (Oyagüe et al., 2009). Therefore, adhesive failures were seen in groups with lower strengths as the n‐HAP groups.

## 5 CONCLUSION

Within the limitations of this in vitro study, we can conclude that the use of desensitizing agents containing, CPP‐ACP, n‐HAP, or fluoride after laser bleaching can enhance the bond strength of the resin cements to enamel when there is an interval of time between the bleaching process and the bonding process. The chemical composition of the resin cements plays an important role in its bonding strength to enamel. Further in vitro and in vivo studies are requested.

## AUTHORS CONTRIBUTIONS

*Study design, specimen preparation, data tabulation, statistical analysis and results writing*: Mohammed N. Anwar. *Study design, review and introduction, interpretation and results discussion writing*: Ahmad K. ElFadl. *Study design, specimen preparation, review and introduction, methodology execution and writing, interpretation and results discussion writing*: Aya E. Samaha.

## Data Availability

The data that support the findings of this study are available from the corresponding author upon reasonable request.
